# Association rule mining and network analysis of the evolving comorbidity patterns in HIV inpatients in Baise, China

**DOI:** 10.3389/fpubh.2026.1717479

**Published:** 2026-03-06

**Authors:** Lihong Zhao, Liuying Tang, Xu Yang, Suren Rao Sooranna, Qiuping Li, Huiying Tan, Huina Guo

**Affiliations:** 1 Faculty of Nursing, Youjiang Medical University for Nationalities, Baise, China; 2 Department of Infectious Diseases, Baise People's Hospital, Baise, China; 3 Faculty of Medicine, Department of Metabolism, Digestion and Reproduction, Imperial College London, London, United Kingdom; 4 Cardiac Intensive Care Unit, Department of Cardiology, Affiliated Hospital of Youjiang Medical University for Nationalities, Baise, China; 5 School of Basic Medical Sciences, Youjiang Medical University for Nationalities, Baise, China

**Keywords:** Apriori algorithm, Baise, comorbidity patterns, dynamic changes, HIV inpatients, network diagram

## Abstract

With the widespread use of antiretroviral therapy, human immunodeficiency virus (HIV) infection is considered to be a manageable chronic disease, but it is accompanied by an increased burden of comorbidities. Baise is an area characterized by a high incidence of HIV infection in Guangxi, China. However, research on its comorbidity patterns is limited. This study aims to clarify the burden, patterns, network features, and temporal evolution of comorbidities among HIV inpatients in Baise. We collected electronic medical records from 3,294 HIV patients hospitalized at Baise People’s Hospital between January 2019 and August 2024. The Apriori algorithm was employed to extract association rules between diseases, while Gephi was utilized to construct comorbidity social network diagrams of the data. The findings revealed that 99.48% of patients presented with two or more comorbidities, with a median of 9 comorbidities per patient. Notably, the median number of comorbidities peaked at 11–12 during 2021–2022, subsequently decreasing to 7 in 2023–2024. The comorbidity patterns transitioned from (B20 + B37 → B99) in 2019 to (E46 + B20 → E87 + D64) in 2021 and ultimately evolved into (J18 + E87 → E46) by 2023. Social network analysis indicated that electrolyte imbalances (E87), HIV-related infections (B20) and candidiasis (B37) served as the core disease nodes within the network, displaying close connections with numerous other disease nodes. The identified specific comorbidity patterns can serve as early warnings and screening tools in clinical practice and they underscore the necessity for multidisciplinary care for HIV patients.

## 1 Introduction

Since the first case of acquired immunodeficiency syndrome (AIDS) was reported in 1981, infections with human immunodeficiency virus (HIV) have emerged as a significant global public health concern. Antiretroviral treatment (ART) has transformed AIDS into a manageable chronic disease, significantly enhancing life expectancy (1). However, the increased lifespan has led to a rise in the burden of comorbidities experienced by HIV infected patients (2–4). Consequently, in the era of ART, effectively managing multiple comorbidities has become a substantial challenge for clinicians and the healthcare system.

Comorbidities are conditions that coexist with a disease of interest (5). Multiple comorbidities typically signify the presence of two or more chronic conditions, excluding a disease of interest (6). However, the definition and scope of multiple comorbidities are not consistently applied in HIV populations (7). This inconsistency hinders the comparability of different studies in HIV-related research and clinical planning efforts. In this study, multiple comorbidities were defined as the presence of two or more diagnosed conditions in addition to HIV infection, encompassing both HIV-related complications (e.g., opportunistic infections) and common chronic conditions (e.g., metabolic diseases) (8). A North American study found that the percentage of ART patients with chronic diseases, such as hypertension, diabetes and kidney disease, rose from 8.2 to 22.4% between 2000 and 2009 (6). Similarly, a study of 8,490 HIV patients in South Carolina also showed an increase in multiple comorbidities from 6.6 to 23.6% between 2005 and 2016 (7). In developing countries such as Brazil, 63% of HIV patients over 50 years old suffered from multiple comorbidities, which was significantly higher than that of the general population within the same age group (9). Multiple comorbidities can significantly influence the quality of life of a patient and increase healthcare costs. Studies indicated that the prevalence of multiple comorbidities among people with HIV is increasing, making clinical management more difficult (6, 10). Multiple comorbidities such as nonalcoholic fatty liver disease (NAFLD) and metabolic syndrome, interact with HIV to exacerbate the chronic inflammation, immune dysfunction and metabolic disorders associated with AIDS, ultimately affecting clinical prognosis (11, 12). Despite these observations, evidence that extends beyond disease prevalence to characterize disease combinations, their association rules, and the evolution of such patterns over time in routine care remains limited.

By the end of 2023, Guangxi had approximately 115,000 people living with AIDS, with a total of 189,000 reported infection cases and 74,000 deaths (13). This region is recognized as a high-incidence area for HIV infection in China, and it predominantly affects rural populations (14). Among the new cases reported in Guangxi in 2023, 66.2% were aged 50 and above, 73.4% were from rural areas and 92.7% were transmitted through heterosexual intercourse (15). Baise City, located in western Guangxi, has a large rural population, a significant number of migrant workers, and it is situated on the border. These factors can exacerbate HIV infection rates. A substantial number of AIDS cases are diagnosed at advanced stages in Baise (16, 17). Late diagnosis frequently results in delayed immune recovery and an increased incidence of opportunistic infections, thereby exacerbating the burden of comorbidities (18). Most studies in Baise have focused on HIV incidence factors and public health interventions. However, research on comorbidity patterns remains limited, especially concerning frequent disease combinations, association rules, and their temporal evolution in HIV inpatients (19).

This study analyzed HIV inpatient records from the high-incidence areas of Baise to identify frequent disease combinations, uncover association rules, and visualize evolving comorbidity structures over time. The findings could clarify regional specificities and guide multidisciplinary care for HIV-related comorbidities.

## 2 Materials and methods

### 2.1 Data sources

Data from electronic medical records were collected from HIV inpatients registered with the Hospital Information System of Baise People’s Hospital between January 2019 and August 2024. The protocol was approved by the Ethics Committee of Youjiang Medical University for Nationalities (Approval No. 2023122639). All patient data were de-identified by removing direct identifiers and replacing them with research-specific IDs to ensure anonymity. The core variables included age, gender, residence, number of hospitalizations, length of hospital stay and comprehensive disease diagnosis records. The initial sample comprised of 3,294 cases, encompassing diseases such as candidiasis, anemia, pneumonia, hypoproteinemia and electrolyte disturbances. A thorough inspection of the data revealed that no key variables were missing across all the cases included in the study. The original records of disease diagnoses for patients comprised 2017 entries, all of which were coded according to the International Classification of Diseases, 10th Revision (ICD − 10). To mitigate data sparsity issues arising from excessively detailed subcategory codes, we retained the core hierarchy of ICD − 10 codes (the first letter followed by two digits). Following this aggregation, 613 independent disease classification variables were generated, thereby establishing the foundational dataset for association rule analysis.

#### 2.1.1 Exclusion criteria for HIV comorbidities

Mental disorders, external injuries, and congenital diseases were excluded from the definition of HIV comorbidity. Mental disorders were specifically excluded because they are not routinely screened or uniformly documented in inpatient electronic medical records. This results in high rates of missed diagnoses and inconsistent coding across departments. Including mental disorders could lead to misclassification, thereby compromising the stability and comparability of subsequent analyses.

#### 2.1.2 Clinical definitions and proxies for HIV disease stage

This study employed several clinical terms to describe the progression of HIV disease. “Late diagnosis” refers to patients diagnosed with HIV at a stage where they already exhibit clinically significant HIV-related illnesses, such as opportunistic infections or AIDS-defining conditions, as documented in discharge diagnoses (20, 21). “Advanced HIV disease” (AHD) could not be defined using CD4 counts due to the unavailability of these data in this study. Instead, we used a clinical proxy based on the WHO staging system. Patients with WHO stage 3 or stage 4 recorded in their discharge diagnoses were classified as having AHD (22, 23). WHO stage assignment was derived from aggregated ICD − 10 codes by mapping diagnoses to stage-defining conditions according to WHO clinical staging guidance (22, 23). “Late immune response” (or “delayed immune recovery”) describes a slow or incomplete recovery of immune function following antiretroviral therapy (24). However, due to the lack of longitudinal laboratory follow-up and treatment history, immune recovery could not be quantified in this study. Therefore, it is discussed descriptively rather than treated as a measurable endpoint (24).

### 2.2 Research methods

#### 2.2.1 Association rule mining

Association rule mining (ARM) is a data mining technique used to determine frequency patterns and associative features among variables within a dataset. In healthcare analytical studies, the Apriori algorithm and its variants can be employed to uncover association rules from large-scale health data, thereby providing valuable support for clinical monitoring, disease prevention and control (25, 26). ARM is particularly well-suited for analyzing inpatient ICD-10 diagnosis data, which is characterized by high dimensionality, sparsity, and the frequent co-occurrence of multiple diseases. By not relying on prior assumptions, ARM facilitates the identification of frequent higher-order comorbidity combinations. Unlike traditional methods such as cross-sectional surveys, regression, and cluster analyses, which often overlook complex interactions among multiple diseases and rely on predefined hypotheses, the Apriori algorithm automatically uncovers the frequency of disease combinations and strong association rules from extensive datasets (7, 27). Understanding frequent disease combination patterns in HIV patients was deemed crucial for developing strategies to prevent multimorbidity, optimize treatments and improve health outcomes (4). The Apriori algorithm is divided into two steps: (1) finding frequent item sets and (2) constructing rules from frequent item sets. Association rules are typically expressed in the form X → Y, where X represents the antecedent condition and Y denotes the consequent outcome (28). The strength of association rules is generally assessed using three critical metrics: support, confidence and lift. Support measures the frequency of the simultaneous occurrence of the antecedent and the consequence as a percentage of the entire dataset. Confidence and lift represent the strength of the association. Strong association rules can be filtered by establishing two thresholds: minimum support and confidence. A lift value greater than 1 indicates a clear directional association in the rule X → Y. This study employed the Apriori algorithm and social network analysis to extract high-frequency disease combinations from ICD-10 diagnostic diseases prominent in Baise, thereby reflecting regional specificity.

#### 2.2.2 Comorbidity social network analysis

The Gephi 0.9.2 software was utilized to construct the comorbidity network of HIV inpatients. While ARM emphasizes local and rule-based disease associations, network analysis offers a complementary perspective by situating these associations within the broader comorbidity framework (29, 30). Network diagrams were employed to enhance the visualization of disease co-occurrence and connection strength, thereby revealing the unique comorbidity structure among HIV patients. In this study, the nodes represented the disease categories (e.g., B37 for candidiasis). The edges signified the co-occurrence associations between two diseases, their weight represented the frequency of co-occurrence between two disease combinations. The widths of the edges were proportional to the weight values, such that a higher weight corresponded to a thicker edge, indicating a stronger correlation between the nodes.

For network visualization, co-occurrence edges were ranked by weight in descending order. High-weight edges were progressively added until each network contained approximately 24 to 34 disease nodes. This approach was consistently applied across different time periods to balance interpretability and structural comparability, while avoiding excessive sparsity or overcrowding. The resulting sets of nodes formed the basis of the comorbidity networks presented in the results section.

#### 2.2.3 Statistical analysis

Statistical analysis was conducted using SPSS version 26.0. Given that the measurement data did not conform to a normal distribution, the results were expressed as medians with 25 and 75% quartiles [M (P25, P75)]. Count data were presented as percentages [n (%)]. The Mann–Whitney U test was employed for comparisons involving binary variables. In contrast, the Kruskal-Wallis H test was used to assess the number of comorbidities across different groups for multi-categorical variables. We also employed Dunn and Kolmogorov–Smirnov tests for analysis of our data. In all cases, a *p*-value of less than 0.05 was considered statistically significant. The Apriori algorithm was executed using R version 4.2.2 (Arules software package) and IBM SPSS Modeler version 26.0 was used to extract comorbidity association rules. The parameters were set with a minimum support of 0.20, confidence of 0.20, lift ≥1.0, and a maximum item set length of 4. These parameters were selected to balance clinical interpretability, computational efficiency, and data denoising in our dataset. A support threshold of 0.20 was applied to capture frequent comorbidities (≥20% of cases), thereby enhancing the generalizability of findings while excluding rare associations. This approach aligns with methods used in COVID-19 symptom mining, where low thresholds have successfully identified key patterns (31). A confidence level of 0.20 facilitated the exploratory detection of clinically valuable association rules, consistent with methodologies employed in temporal multimorbidity analyses derived from electronic health records (32). To exclude negative correlations, only rules with a lift value of 1.0 or greater were retained. Specifically, a lift value of 1 indicates statistical independence, whereas a lift value greater than 1 represents a positive correlation. Finally, a maximum item set length of 4 was chosen to focus on interpretable combinations of 2 to 4 diseases. This constraint prevents rule explosion in high-dimensional data, a strategy supported by prior research in large-scale disorder pattern mining (33). A comorbidity social network diagram was constructed with Gephi version 0.9.2, and we analyzed the dynamic alterations in comorbidity patterns over various years.

To evaluate temporal changes across admission periods (2019–2020, 2021–2022, and 2023–2024), we conducted several comparative analyses. First, we assessed differences in the number of comorbidities between the three periods using the Kruskal-Wallis test, followed by Dunn’s post-hoc comparisons with Holm adjustment; effect size (ε^2^) was also reported. Second, we evaluated comorbidity association patterns across the admission periods by calculating the Jaccard similarity of the top 20 support-ranked unidirectional comorbidity association patterns. Finally, for each admission period, we constructed an undirected weighted comorbidity network based on the top 20 edges ranked by co-occurrence weight, and we analyzed network-level characteristics across the periods.

## 3 Results

### 3.1 Comorbidity burden in HIV inpatients

**Figure 1:**
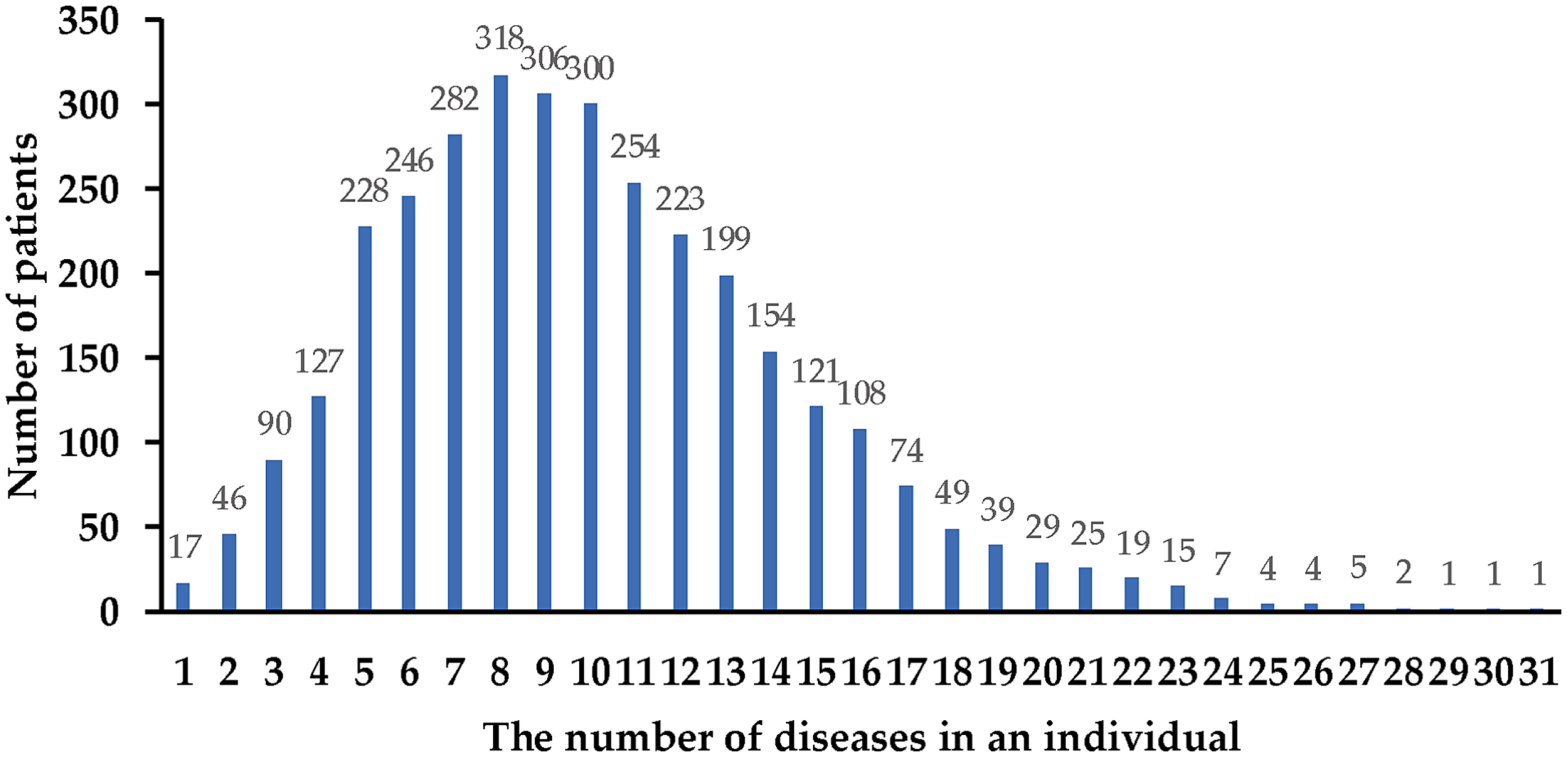
The number of different associated diseases found in HIV inpatients.

A total of 613 diseases were diagnosed among the 3,294 HIV inpatients in this study. Only 17 patients (0.52%) were diagnosed with a single disease, while 3,277 patients (99.48%) presented with two or more diseases, exhibiting multiple comorbidities (Figure 1). Most of patients exhibited between 5 to 12 diseases, with 8 diseases being the most frequently observed (9.65%, *n* = 318). A notable subset of these patients (3.28%, *n* = 113) had more than 20 diseases. The median number of comorbidities among patients was 9. The Kolmogorov–Smirnov test indicated that the distribution was skewed, with an interquartile range from 7 (25th percentile) to 13 (75th percentile).

**Figure 2:**
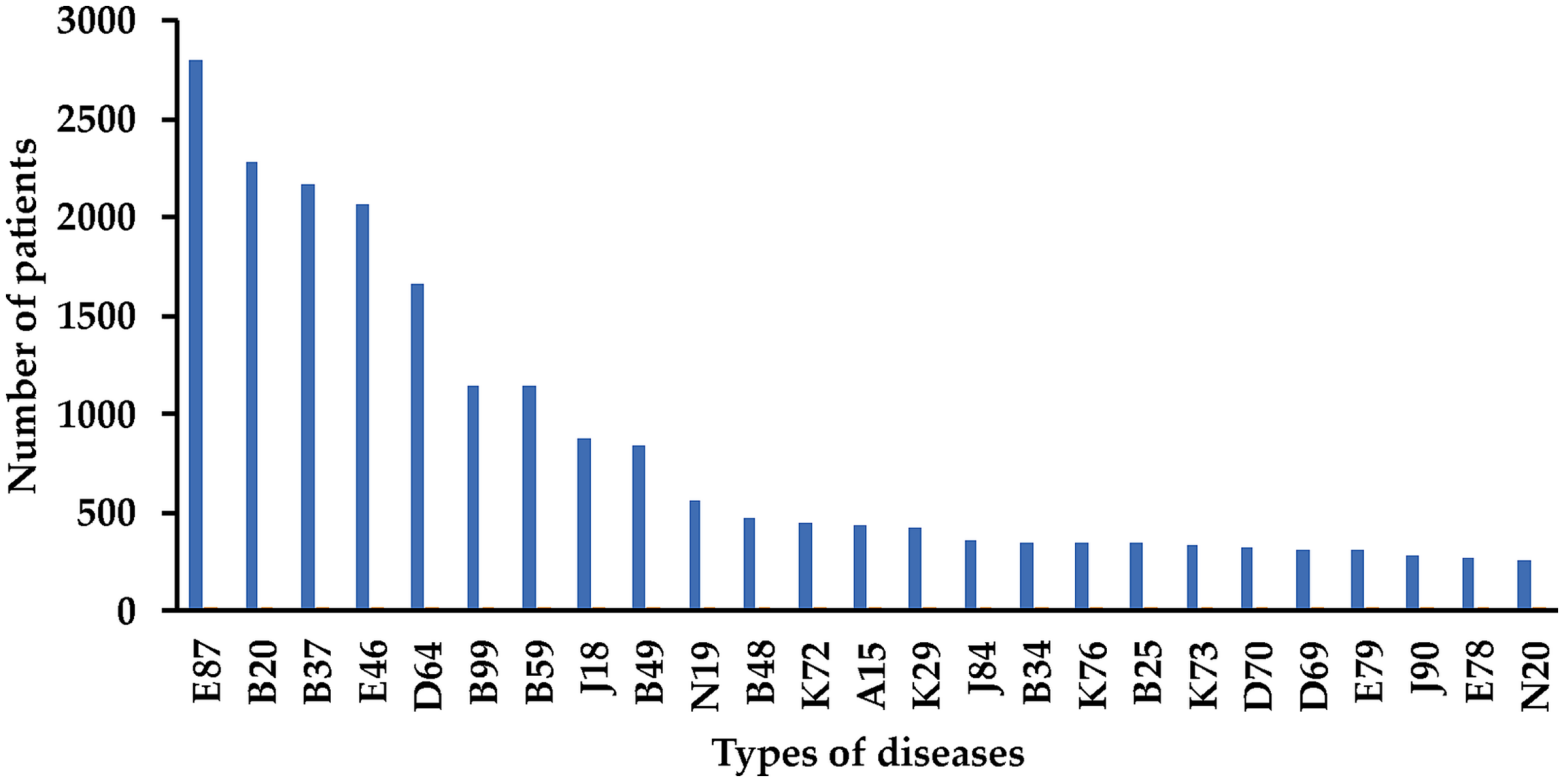
The top 25 prevalence of diseases found among our HIV inpatients. E87, Electrolyte disorders; B20, multiple infections and tuberculosis due to HIV disease; B37, candidiasis; E46, hypoproteinemia; D64, anemia; B99, bacterial infection; B59, pneumocystosis; J18, pneumonia; B49, fungal infection; N19, renal failure; B48, mycosis; K72, hepatic failure; A15, pulmonary tuberculosis; K29, gastritis and duodenitis; J84, interstitial pneumonia; B34, viral infection; K76, liver diseases (e.g., fatty liver disease); B25, cytomegaloviral disease; K73, chronic hepatitis; D70, leukopenia; D69, thrombocytopenia; E79, hyperuricemia; J90, pleural effusion; E78, hyperlipidemia; N20, renal calculus.

The top 25 prevalent diseases among HIV inpatients are detailed in Figure 2. Specifically, the top 3 prevalences of diseases were electrolyte disorders (E87, 85.57%), HIV-related infections, including tuberculosis (B20, 69.70%) and candidiasis (B37, 65.91%).

### 3.2 Comparison of the comorbidity numbers across subgroups in our cohort

Among the 3,277 patients in the comorbidity cohort, there were 2,381 males (72.66%) and 896 females (27.34%). Additionally, 2,579 patients (78.70%) were from rural areas, while 698 patients (21.30%) were from urban areas (Table 1). The average length of hospital stay was 10.9 ± 6.5 days, and the mean number of hospitalizations was 2.1 ± 2.0. A total of 1787 patients (54.53%) in the comorbidity cohort met the WHO stage 3/4 proxy definition for advanced HIV disease (Table 1). No significant differences were observed in the number of comorbidities among various subgroups, including gender, age, residence and the number/length of hospitalizations. Notably, the median number of comorbidities was significantly higher in patients admitted in 2021 (11) and 2022 (12) compared to other years (*H* = 370.097, *p* < 0.001). Collectively, these two years accounted for nearly 40% of all hospitalizations. By 2024, the median number of comorbidities had declined to 7.00. The results of the pairwise comparisons using Dunn’s t-test with Holm adjustment indicated that the differences in the number of comorbidities among patients across each annual group were statistically significant, except for the comparisons between 2019–2020, 2021–2022, and 2023–2024. The comorbidity numbers differed significantly across the admission periods (*p* ≤ 0.01). Pairwise comparisons indicated that there were significant differences among all three periods, as shown in Supplementary Table S1.

**Table 1:** Comparison of the number of comorbidities among patients in different subgroups.

Subgroups	Number of patients	Percentage (%)	M (P25, P75)	Statistic	*p* value
Gender				-1.085^a^	0.278
Male	2,381	72.66	10.000 (7.0, 13.0)		
Female	896	27.34	9.000 (7.0, 12.0)		
Place of residence				-1.288^a^	0.198
Rural	2,579	78.70	10.000 (7.0, 13.0)		
Urban	698	21.30	9.000 (7.0, 12.0)		
Admission years				370.097^b^	< 0.001
2019	640	19.53	9.00 (7.0, 12.0)		
2020	560	17.09	10.00 (7.0, 12.0)		
2021	644	19.65	11.00 (8.0, 14.0)		
2022	596	18.19	12.00 (8.0, 15.0)		
2023	523	15.96	8.00 (6.0, 10.0)		
2024	314	9.58	7.00 (5.0, 9.0)		
Number of hospitalizations				-1.282^a^	0.200
1	1746	53.28	10.00 (7.0, 13.0)		
≥ 2	1,531	46.72	9.00 (7.0, 13.0)		
Length of hospital stay (Day)				2.219^b^	0.528
1–9	1,527	46.60	9.00 (7.0, 13.0)		
10–19	1,462	44.61	10.00 (7.0, 13.0)		
20–29	248	7.57	9.00 (7.0, 13.0)		
≥30	40	1.22	10.00 (5.3, 12.8)		
Age				0.686^b^	0.876
0–20	15	0.46	10.00 (8.0, 13.0)		
20–40	577	17.61	10.00 (7.0, 13.0)		
40–60	1,646	50.23	9.00 (7.0, 13.0)		
60–90	1,039	31.71	9.00 (7.0, 13.0)		
WHO clinical stage					
Stage 1	1,382	42.17			
Stage 2	108	3.30			
Stage 3	728	22.22			
Stage 4	1,059	32.32			
Total/each subgroup	3,277	100.00			

M (P25, P75) represents median numbers of comorbidities with 25 and 75% quartiles. a is the statistic Z value of the Mann–Whitney U test. b is the statistic H value of the Kruskal-Wallis H test.

### 3.3 Comorbidity pattern mining based on the Apriori algorithm in our cohort

#### 3.3.1 Overall analysis of frequent item sets

This study utilized the Apriori algorithm to analyze frequency of item sets and association rules. The parameters were set as follows: minimum confidence = 0.20, minimum lift = 1.0, and maximum itemset length = 4. The frequent itemset analysis was conducted on all HIV inpatients with comorbidities (Table 2). The results indicated that the most common two-disease combinations included Candidiasis and Electrolyte disorders (B37 + E87, support = 0.43), Hypoproteinemia and Electrolyte disorders (E46 + E87, support = 0.41), and HIV-related infections and Candidiasis (B20 + B37, support = 0.40). A notable three-disease combination was Hypoproteinemia, Candidiasis and Electrolyte disorders (E46 + B37 + E87, support = 0.30). Among the four-disease combinations, Hypoproteinemia, Candidiasis, Electrolyte disorders and Anemia (E46 + B37 + E87 + D64, support = 0.20) emerged as the most significant. The high-frequency comorbidities in this study indicated that the disruption of metabolic homeostasis significantly contributed to the comorbidity burden in HIV-infected patients, as described by Yang et al. (2021) (7).

**Table 2:** Frequent item sets of comorbidities in the studied cohort.

Frequent items	Support	Length of items
(E87, B37)	0.43	2
(E87, E46)	0.41	2
(B20, B37)	0.40	2
(E46, B37)	0.38	2
(D64, E87)	0.36	2
(E87, B20)	0.36	2
(D64, E46)	0.35	2
(D64, B37)	0.34	2
(E46, B20)	0.32	2
(D64, B20)	0.29	2
(B99, B37)	0.28	2
(B99, B20)	0.27	2
(E87, B99)	0.24	2
(B99, E46)	0.23	2
(E87, E46, B37)	0.30	3
(D64, E87, E46)	0.28	3
(E87, B20, B37)	0.28	3
(D64, E87, B37)	0.26	3
(D64, E46, B37)	0.25	3
(E87, E46, B20)	0.25	3
(E46, B20, B37)	0.24	3
(B99, B20, B37)	0.22	3
(D64, E87, B20)	0.22	3
(D64, B20, B37)	0.22	3
(D64, E46, B20)	0.21	3
(D64, E87, E46, B37)	0.20	4

E87, Electrolyte disorders; B20, Multiple infections and tuberculosis due to HIV disease; B37, Candidiasis; E46, Hypoproteinemia; D64, Anemia; B99, Bacterial infection.

#### 3.3.2 Annual dynamics analysis of frequent item sets

To further investigate the temporal dynamics of comorbidity patterns, patients were grouped into three admission periods: 2019–2020, 2021–2022 and 2023–2024. We identified high-frequency disease sets (2–4 items) within each period and selected the top three ones by support. Comorbidity patterns shifted notably over time: metabolic-nutritional disorders E46 and E87, and opportunistic infection B37 were the core ones in 2019–2020. HIV-related disease (B20) became predominant in 2021–2022 and pneumonia (J18) with electrolyte disorders (E87) emerged as dominant in 2023–2024 (Table 3).

**Table 3:** Frequent item sets of comorbidities in different time periods of patient admission.

Admission years	Items	Support	Length of items
2019, 2020	(E46, B37)	0.46	2
	(B20, B37)	0.43	2
	(E87, B37)	0.43	2
	(E87, E46, B37)	0.33	3
	(D64, E46, B37)	0.32	3
	(E87, D64, E46)	0.30	3
	(E87, D64, E46, B37)	0.24	4
	(E87, E46, B20, B37)	0.22	4
2021, 2022	(B20, B37)	0.58	2
	(B20, E87)	0.54	2
	(B37, E87)	0.51	2
	(B20, B37, E87)	0.41	3
	(B20, E87, E46)	0.36	3
	(E46, B37, E87)	0.34	3
	(B20, B37, E87, E46)	0.27	4
	(B20, E87, E46, D64)	0.25	4
	(B20, B37, E87, B99)	0.24	4
2023, 2024	(J18, E87)	0.38	2
	(B37, J18)	0.32	2
	(B37, E87)	0.32	2
	(B37, E87, J18)	0.24	3
	(E46, J18, E87)	0.20	3

E87, Electrolyte disorders; B20, Multiple infections and tuberculosis due to HIV disease; B37, Candidiasis; E46, Hypoproteinemia; D64, Anemia; B99, Bacterial infection.

#### 3.3.3 Overview of strong association rule mining

The analysis of association rules revealed varying strengths of association between antecedent and consequent disease combinations (Table 4). High-lift rules, such as (B20 + B37) → (B99) (lift = 1.61) and (E46 + B37 → E87 + D64) (lift = 1.48), implied strong positive correlations between the above antecedent and consequent disease combinations. Furthermore, the rules with high confidence, including (B20 + B99) → (B37) (confidence = 0.81) and (E46 + B37 + D64) → (E87) (confidence = 0.81), indicated a high probability of the consequent disease giving rise to the antecedent disease. The leverage of all rules was positive, demonstrating that the co-occurrence was more frequent than expected under independence. Noteworthy examples, such as (D64 + E87) → (E46) (leverage = 0.08), illustrated the relatively strong associations of the above disease combinations. In addition, the rules with high conviction, including (B37 + B99) → (B20) (convictio*n* = 2.30), further reinforced the strong associations between these disease combinations.

**Table 4:** Strong association rules for comorbidities in our cohort of inpatients.

Antecedent	Consequent	Support by antecedent	Support by consequent	Support	Confidence	Lift	Leverage	Conviction
(B99)	(B20, B37)	0.34	0.40	0.22	0.65	1.61	0.08	1.69
(B20, B37)	(B99)	0.40	0.34	0.22	0.55	1.61	0.08	1.45
(B99, B37)	(B20)	0.28	0.53	0.22	0.79	1.51	0.07	2.30
(B99)	(B20)	0.34	0.53	0.27	0.79	1.51	0.09	2.30
(B20)	(B99)	0.53	0.34	0.27	0.51	1.51	0.09	1.36
(B20)	(B99, B37)	0.53	0.28	0.22	0.42	1.51	0.07	1.24
(D64, E87)	(E46, B37)	0.36	0.38	0.20	0.56	1.48	0.07	1.42
(E46, B37)	(D64, E87)	0.38	0.36	0.20	0.54	1.48	0.07	1.38
(D64, E87, B37)	(E46)	0.26	0.54	0.20	0.78	1.45	0.06	2.10
(D64, B37)	(E87, E46)	0.34	0.41	0.20	0.59	1.45	0.06	1.45
(E87, E46)	(D64, B37)	0.41	0.34	0.20	0.50	1.45	0.06	1.31
(E46)	(D64, E87, B37)	0.54	0.26	0.20	0.38	1.45	0.06	1.19
(D64, E87)	(E46)	0.36	0.54	0.28	0.77	1.43	0.08	1.99
(E46)	(D64, E87)	0.54	0.36	0.28	0.52	1.43	0.08	1.32
(D64, B20)	(E46)	0.29	0.54	0.21	0.74	1.38	0.06	1.79
(E46)	(D64, B20)	0.54	0.29	0.21	0.40	1.38	0.06	1.18
(D64, B37)	(E46)	0.34	0.54	0.25	0.73	1.37	0.07	1.73
(E87, E46, B37)	(D64)	0.30	0.50	0.20	0.69	1.37	0.05	1.59
(E46)	(D64, B37)	0.54	0.34	0.25	0.47	1.37	0.07	1.24
(D64)	(E87, E46, B37)	0.50	0.30	0.20	0.41	1.37	0.05	1.19

E87, Electrolyte disorders; B20, Multiple infections and tuberculosis due to HIV disease; B37, Candidiasis; E46, Hypoproteinemia; D64, Anemia; B99, Bacterial infection.

#### 3.3.4 Annual dynamic changes in comorbidity association patterns

To understand the annual dynamic changes in comorbidity association patterns, the Apriori algorithm was employed to identify the top two association rules by lift in each time period (Table 5). The comorbidity patterns exhibited a temporal shift and were initially driven by infections (B20 + B37 → B99) in 2019, transitioning to a coexistence of infections and metabolic disorders (E46 + B20 → E87 + D64) in 2021 and ultimately evolving to pneumonia coupled with metabolic imbalance (J18 + E87 → E46) in 2023. The patterns of comorbidity associations varied across the three admission periods, as indicated by the Jaccard similarity coefficients (Supplementary Table S2).

**Table 5:** Strong association rules for comorbidities in different time periods in the studied cohort.

Admission years	Antecedent	Consequent	Support by antecedent	Support by consequent	Support	Confidence	Lift	Leverage	Conviction
2019, 2020	(B20, B37)	(B99)	0.43	0.38	0.24	0.56	1.46	0.08	1.40
	(B99)	(B20, B37)	0.38	0.43	0.24	0.63	1.46	0.08	1.54
	(E46, B37)	(E87, D64)	0.46	0.36	0.24	0.52	1.44	0.07	1.33
	(E87, D64)	(E46, B37)	0.36	0.46	0.24	0.66	1.44	0.07	1.61
2021, 2022	(B20, E46)	(E87, D64)	0.43	0.42	0.25	0.60	1.42	0.08	1.44
	(E87, D64)	(B20, E46)	0.42	0.43	0.25	0.61	1.42	0.08	1.46
	(E87, B49)	(E46)	0.25	0.58	0.20	0.80	1.39	0.06	2.14
	(E46)	(E87, B49)	0.58	0.25	0.20	0.35	1.39	0.06	1.15
2023, 2024	(E46)	(J18, E87)	0.37	0.38	0.20	0.55	1.43	0.06	1.37
	(J18, E87)	(E46)	0.38	0.37	0.20	0.52	1.43	0.06	1.33
	(D64)	(E46)	0.40	0.37	0.21	0.52	1.42	0.06	1.32
	(E46)	(D64)	0.37	0.40	0.21	0.57	1.42	0.06	1.39

E87, Electrolyte disorders; B20, Multiple infections and tuberculosis due to HIV disease; B37, Candidiasis; E46, Hypoproteinemia; D64, Anemia; B99, Bacterial infection.

### 3.4 Comorbidity social network analysis

#### 3.4.1 Overview of comorbidity association network diagram

**Figure 3:**
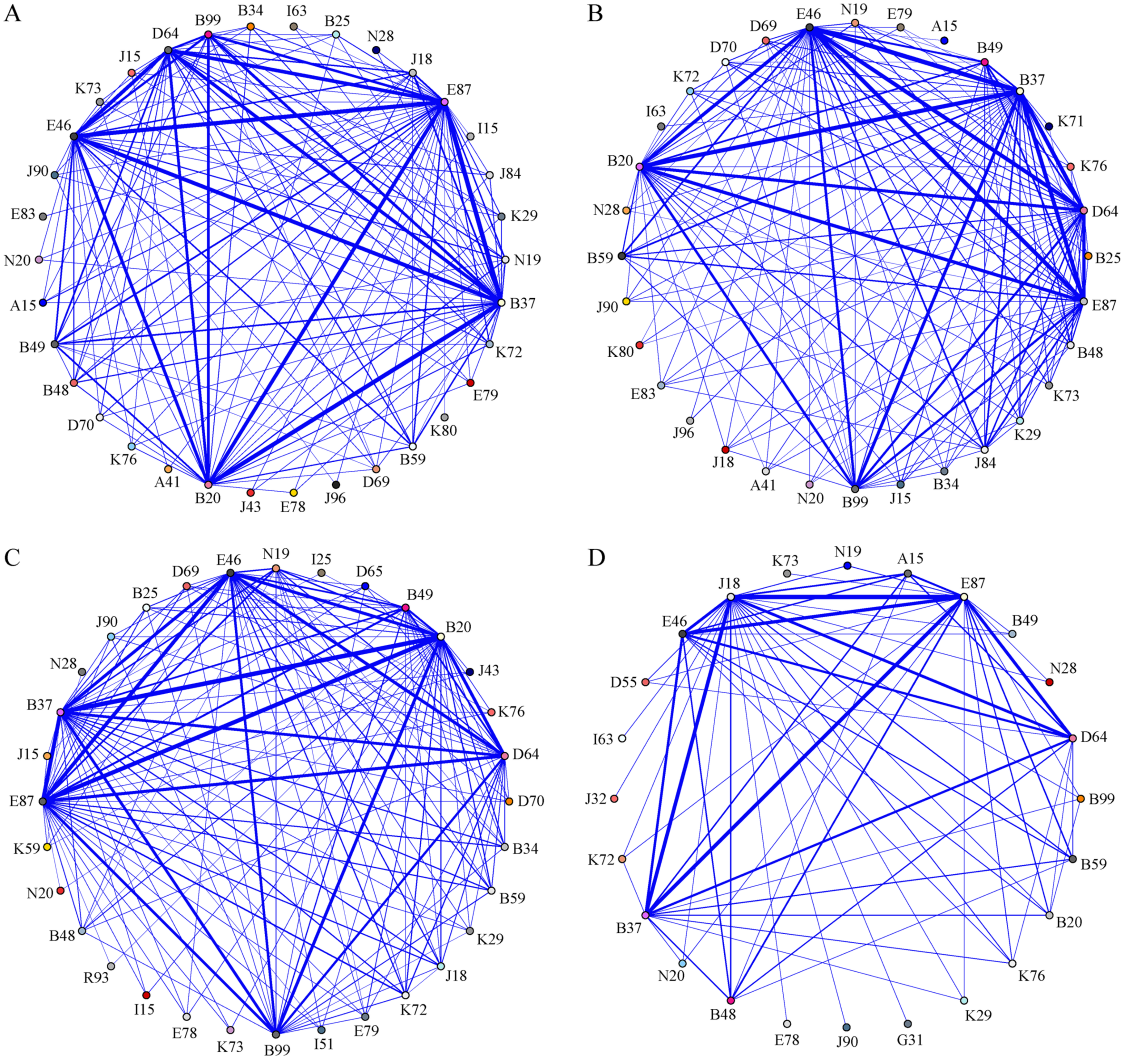
Comorbidity association network diagrams. **(A)** Overview of the comorbidity association network diagram, **(B)** A comorbidity association network diagram of 2019–2020, **(C)** A comorbidity association network diagram of 2021–2022, **(D)** A comorbidity association network diagram of 2023–2024. E87, Electrolyte disorders; B20, Multiple infections and tuberculosis due to HIV disease; B37, Candidiasis; E46, Hypoproteinemia; D64, Anemia; B99, Bacterial infection; B59, Pneumocystosis; J18, Pneumonia; B49, Fungal infection; N19, Renal failure; B48, Mycosis; K72, Hepatic failure; A15, Pulmonary tuberculosis; K29, Gastritis and duodenitis; J84, Interstitial pneumonia; B34, Viral infection; K76, Liver diseases (e.g., fatty liver disease); B25, Cytomegalo-viral disease; K73, Chronic hepatitis; D70, Leukopenia; D69, Thrombocytopenia; E79, Hyperuricemia; J90, Pleural effusion; E78, Hyperlipidemia; N20, Renal calculus, J15, Bacterial pneumonia; N28, Acquired cyst of the kidney; I63, Cerebral infarction; J43, Emphysema; K80, Cholelithiasis/Gallstones; I15, Secondary hypertension; J32, Chronic sinusitis; J96, Respiratory failure; A41, Sepsis; E83, Hypocalcemia; D55, Glucose-6-phosphate dehydrogenase deficiency; I51, Myocardial damage; K59, Intestinal dysbiosis; D65, Coagulopathy; K71, Drug-induced liver injury; I10, Primary hypertension.

The comorbidity association network diagram illustrated several strong disease node combinations (Figure 3A). Node combinations with a weight of ≥ 1,000 included B37 and E87, E87 and E46, B37 and B20, B37 and E46, E87 and D64, E87 and B20, E46 and D64, B37 and D64, and E46 and B20. Detailed weighted value information is provided in Supplementary Table S3. Additionally, node importance was quantitatively assessed using degree, weighted degree, and betweenness centrality, in conjunction with edge weight distribution (Table 6). Diseases such as B37, E87, E46, and B20 ranked among the highest nodes across all centrality metrics. This indicates their central roles and extensive connectivity within a network characterized by moderate to high density (Supplementary Table S4 and Table 6). The social network diagram clearly illustrates that, nodes such as B37, E87, E46, B20 and D64, formed the central hub of the network. These nodes displayed close connections with numerous other disease nodes, emphasizing their pivotal role in the progression of comorbidities.

**Table 6:** Node-level centrality metrics of hub diseases across different admission periods.

Period	ICD-10	Degree	Weighted degree	Betweenness centrality
2019–2020	B37	30	4,890	0.585
2019–2020	E46	29	4,614	0.270
2019–2020	E87	26	4,150	0.028
2021–2022	E87	30	5,938	0.620
2021–2022	B20	29	5,823	0.214
2021–2022	B37	28	5,464	0.032
2023–2024	J18	22	1996	0.518
2023–2024	E87	18	1995	0.415
2019–2024	E87	33	12,678	0.646
2019–2024	B37	28	11,837	0.195
2019–2024	B20	24	9,703	0.030

B37, Candidiasis; E46, Hypoproteinemia; E87, Electrolyte disorders; B20, Multiple infections and tuberculosis due to HIV disease; J18, pneumonia.

#### 3.4.2 Annual comorbidity association network diagrams

To visualize the annual evolution of comorbidity patterns, social network diagrams were plotted based on disease co-occurrence weights (Figures 3B–D). The network structures exhibited distinct stage-specific characteristics across different time-periods: (1) 2019–2020: This stage was predominantly characterized by opportunistic infections. The most significant link was observed between Candidiasis (B37) and Hypoproteinemia (E46) (weight = 553), followed by the association between HIV-related infections (B20) and Candidiasis (B37) (weight = 520). Consistent with these strong pairwise associations, B37 and E46 exhibited higher weighted degree values (Table 6). These network metrics confirmed their central positions within the network during this period, indicating early associations between infections and metabolic disorders. (2) 2021–2022: The comorbidity pattern evolved into a ‘dual-core’ structure, featuring both infections and metabolic disorders. The link between B20 and B37 significantly strengthened (weight = 713). Concurrently, Electrolyte disorders (E87) exhibited stronger associations with multiple diseases, reflecting the synergy between immunosuppression and metabolic abnormalities. Quantitative network metrics demonstrated that E87 exhibited significantly higher degree and betweenness centrality, suggesting its bridging role between infection and metabolic comorbidity groups (Table 6). (3) 2023–2024: A new pattern emerged, dominated by pneumonia (J18) and metabolic comorbidities. The incidence of pneumonia notably increased, and its co-occurrence with Electrolyte disorders (E87) reached a weight of 590. Detailed weighted value information is provided in Supplementary Table S5. Consistent with this pattern, network metrics indicated that J18 showed a high weighted degree and betweenness centrality, thereby reinforcing its central role within the comorbidity network (Table 6). This shift established pneumonia and chronic metabolic issues as the primary reasons for hospitalization of inpatients. Network-level characteristics derived from the top 20 weighted edges revealed variations in the number of nodes, density, average weighted degree, and clustering coefficient across these admission periods (Supplementary Table S6).

## 4 Discussion

This study revealed a high burden of multiple morbidities among HIV inpatients in Baise, Guangxi. The top 3 prevalent diseases were electrolyte disorders (E87), HIV-related infections, including tuberculosis (B20) and candidiasis (B37). The median number of comorbidities was significantly higher in patients admitted in 2021 and 2022 compared to other years. The comorbidity patterns transitioned from (B20 + B37 → B99) in 2019 to (E46 + B20 → E87 + D64) in 2021 and ultimately evolved into (J18 + E87 → E46) by 2023. These patterns exhibited significant regional characteristics and annual dynamic changes. This study developed a comprehensive analytical framework combining ARM with network visualization. This approach enabled simultaneous identification of frequent comorbidity combinations, strong association rules, and key hub nodes among hospitalized HIV patients.

Specifically, 99.48% of patients had at least two comorbidities, with a median of 9 comorbidities per patient. This proportion was markedly higher than the levels reported in other regions. For instance, the North American AIDS Cohort Collaboration on Research and Design reported that the prevalence of age-related chronic comorbidities among ART-treated populations in the United States ranged from 8.2 to 22.4% (6). In Nanning, the prevalence of comorbidities among HIV inpatients was 71.2%. The following reasons might explain the disparity (34). First, the study focused on hospitalized patients, who in general presented with more severe conditions. HIV inpatients with low immunity were susceptible to multi-infectious diseases (34). Second, this study included both HIV-related complications and comorbidities. Finally, Baise is a high-HIV-prevalence area in western China with a unique epidemiological profile. According to the AIDS epidemic data from 1996 to 2022 in Baise, the majority of cases were among farmers (67.37%) and the Zhuang ethnic group (77.78%) (16). In addition, Baise is close to the borders of Vietnam, and has frequent migration of migrant workers. Limited healthcare services in rural areas can delay the diagnosis and treatment of AIDS, which is consistent with the finding that ineffective surveillance and control strategies promote HIV transmission and the development of comorbidities (35, 36).

There is no significant effect on the number of comorbidities based on gender, age, and residence (*p* > 0.05). This result contrasts with some studies (37). Males and unemployed individuals were reported to be more likely to develop psychiatric comorbidities in a Nigerian study (38). Older HIV patients (≥ 50 years) in Guangxi have been reported to exhibit an elevated risk of mortality, probably affected by age, residence and late time of diagnosis. However, no direct association has been established between these factors and the number of comorbidities (39, 40). This discrepancy may stem from differences in the definitions of comorbidities. While our study specifically focused on somatic diseases and excluded mental illnesses, the Nigerian study incorporated the different psychiatric conditions experienced by the participants. This methodological choice may have resulted in an underestimation of the overall comorbidity burden associated with mental health. Future studies that incorporate standardized mental health assessments and more comprehensive medical records are warranted.

The elevated prevalence of HIV-related infections (B20) and candidiasis (B37) was primarily attributable to the destruction of CD4 + T cells by HIV, thereby leading to reduced immune defenses (41). Pneumonia (39.8%), tuberculosis (35.3%) and candidiasis (28.8%) were the most common opportunistic infections among hospitalized HIV patients in Nanning (34). Cen et al. identified immunosuppression as a core risk factor for multiple opportunistic infections in HIV patients with concurrent Penicillium marneffei infections occurring in Guangxi (42). Furthermore, they noted that these patients often experienced concurrent infections with multiple pathogens, including fungi and bacteria.

Electrolyte imbalance (E87) and hypoproteinemia (E46, 63.20%) were the most prevalent metabolic comorbidities associated with HIV infections. Their high prevalence stems from the multifactorial nature of HIV infection, which includes chronic wasting symptoms, malabsorption and the side effects of treatments (43, 44). Dauphinais et al. defined malnutrition as nutritionally AIDS (N-AIDS), which can increase infection risk by impairing immune cell function (45). This creates a cyclical pattern of malnutrition, immunosuppression and infection. Electrolyte imbalance and anemia were significant prognostic risk factors found in patients with HIV and cryptococcal infection in Guangxi, and this was consistent with the strong association to diseases observed in our study (46). Furthermore, Zhang et al. have suggested that gut microbial dysbiosis in HIV infections can disrupt nutrient absorption, resulting in hypoproteinemia and electrolyte imbalances (47).

Comorbidity social network analysis revealed that E87, B20 and B37 were the core hub nodes, with weights > 1,000. This finding is consistent with the studies conducted by Gama et al. on hospitalized AIDS patients in the Brazilian Amazon region, which indicated that respiratory syndromes, including pneumonia, tuberculosis and malnutrition, served as significant predictors of mortality among this population (48). This result aligns closely with several clinical studies conducted in Guangxi. For instance, Lai et al. found that pneumonia and hypoproteinemia were key predictors of in-hospital mortality in patients with cytomegalovirus infection (49). In addition, Meng et al. reported that patients with multiple opportunistic infections experienced a significantly higher mortality rate, with electrolyte imbalances further complicating treatment (34). Consequently, patients who present with these comorbidities should receive intensive care (50, 51). The risk scoring system may serve as a valuable reference to mitigate mortality risk through multidisciplinary consultations and extended hospitalizations (46).

The comorbidity pattern of hospitalized HIV patients in Baise showed stage evolution between 2019 and 2024, reflecting the combined influence of local epidemiology, healthcare system factors, and external disruptions over time. During 2019 to 2020, opportunistic infections and nutritional/metabolic abnormalities frequently co-occurred, indicating that many patients presented with advanced disease. Delayed diagnosis is common in rural southwestern China, particularly in resource-limited areas such as Baise (40, 52). High population mobility in the border region may further disrupt the continuity of HIV care, thereby raising the risk of late presentation and severe illness (53, 54). In immunocompromised individuals, opportunistic infections often coincide with conditions such as hypoproteinemia and electrolyte disturbances, forming an “infection-metabolic imbalance” profile that is linked to poorer outcomes (36, 42). Between 2021 and 2022, nationwide non-pharmaceutical interventions during the COVID-19 pandemic likely disrupted routine HIV services, resulting in delays in ART initiation, reductions in CD4 counts, and interruptions in follow-up care (55–57). These systemic interruptions may have exacerbated disease severity among patients admitted in Baise, elevating the median number of comorbidities to 11–12. Concurrently, they appear to have increased hospitalizations of individuals with complex multisystemic conditions-particularly malnutrition, electrolyte disorders, and anemia-which often coexist and interact, further complicating clinical management (34, 43). By 2023–2024, the comorbidity pattern had shifted toward pneumonia combined with metabolic disorders, while the median number of comorbidities decreased. This shift can be attributed to two concurrent trends: first, the rising proportion of older rural patients in Guangxi’s HIV population (15); and second, the growing burden of metabolic complications associated with prolonged ART exposure and aging (39, 58). In rural areas, chronic disease management and nutritional support frequently lag behind the expansion of ART, leaving older patients especially susceptible to acute infections due to pre-existing metabolic and nutritional deficits- a key factor contributing to hospital admissions during this period (59–61).

Overall, the evolution of comorbidity patterns among hospitalized HIV patients in Baise is not driven by a single factor but rather by multiple factors at different stages, including the timing of diagnosis, continuity of health services, pandemic disruptions, and population aging. These findings highlight the need in resource-limited areas to not only expand ART coverage but also to strengthen continuity of care, provide early integrated management of opportunistic infections and metabolic disorders, and incorporate chronic comorbidity management into multidisciplinary inpatient care frameworks. Analysis of comorbidity patterns across admission periods revealed temporal evolution in the comorbidity structure of HIV inpatients, reflecting distinct disease profiles over time. Due to the unavailability of CD4 data, advanced HIV disease was defined using a clinical proxy based on WHO stage 3/4 discharge diagnoses (23). This approach may be sensitive to the completeness of coding and documentation practices (22). Consequently, patients classified as WHO stage 1–2 in this study represent individuals without documented stage 3–4 conditions, which does not necessarily confirm early infection or preserved immune function. This proxy definition is aligned with the clinical framing of advanced HIV disease in WHO guidance (22).

Although this study is based on inpatient data from Baise, a high-burden rural area, the core comorbidity evolution patterns it reveals and the key driving factors can be transferable to other global regions with similar epidemiological characteristics. For example, in rural sub-Saharan Africa or border areas of Latin America, delayed diagnosis and population mobility similarly exacerbate the burden of multiple comorbidities (7, 9, 48). To mitigate late diagnosis and the complexities of comorbidities in similar rural and floating populations, it is essential to expand early and accessible HIV testing while ensuring rapid linkage to ART, particularly for individuals who meet the WHO stage 3/4 proxy definition of advanced HIV disease (22). This upstream intervention directly complements the multidisciplinary inpatient management strategies informed by our comorbidity findings. Methodologically, the Apriori algorithm and Gephi network analysis provide scalable tools for extracting dynamic comorbidity rules from electronic health records worldwide, which are adaptable to diverse datasets with minimal adjustments for variations in ICD coding (25, 26). However, regional differences, such as late diagnoses observed in Baise, limit direct extrapolation to well-resourced urban settings (e.g., North America (6)), underscoring the necessity for context-specific adaptations in global HIV strategies. Future studies should validate these association rules in multicenter inpatient cohorts. Integrating routinely collected laboratory indicators, such as CD4 count, viral load, albumin, and electrolyte levels, could improve clinical interpretability. Furthermore, linking comorbidity patterns with outcomes including mortality, readmission, and length of stay may further support the development of risk stratification and targeted intervention strategies.

## 5 Conclusion

Hospitalized HIV patients in a local major hospital in the Guangxi province of China had a high burden of multiple other diseases. Key comorbidities included electrolyte imbalances (E87), HIV-related infections (B20) and candidiasis (B37), which were influenced by the COVID-19 pandemic and ART. These conditions interacted, creating a vicious cycle of weakened immunity, poor nutrition and repeated infections. This study identified specific comorbidity patterns (e.g., (B20 + B99) → (B37)) that can serve as early warnings in clinical practice. The findings emphasize the need for multidisciplinary care for HIV inpatients. Future studies should focus on early intervention strategies which will improve the quality of life for patients as well as reduce healthcare costs.

## Data Availability

The original contributions presented in the study are included in the article/Supplementary material, further inquiries can be directed to the corresponding author.
