# Supplementary material for: Association rule mining and network analysis of the evolving comorbidity patterns in HIV inpatients in Baise, China
Source: Front Public Health. 2026 Mar 6;14:1717479. doi: 10.3389/fpubh.2026.1717479 (PMC13002846; doi:10.3389/fpubh.2026.1717479)
Supplement: Supplementary file 1 [file Table_1.docx]

**Table S1.** Comparison of comorbidity numbers across three admission periods.

| Test | Kruskal-  Wallis H | *P*-value | Effect size ε² | Pairwise comparisons (Holm-adjusted *P*) | | |
| --- | --- | --- | --- | --- | --- | --- |
|  |  |  |  | 2019-2020 vs 2021-2022 | 2019-2020 vs 2023-2024 | 2021-2022 vs 2023-2024 |
| Comorbidity numbers | 358.10 | 1.73 × 10⁻⁷⁸ | 0.109 | 1.21 × 10⁻¹⁹ | 3.57 × 10⁻³¹ | 1.65 × 10⁻⁷⁴ |
