# Supplementary material for: Association rule mining and network analysis of the evolving comorbidity patterns in HIV inpatients in Baise, China
Source: Front Public Health. 2026 Mar 6;14:1717479. doi: 10.3389/fpubh.2026.1717479 (PMC13002846; doi:10.3389/fpubh.2026.1717479)
Supplement: Supplementary file 2 [file Table_2.docx]

**Table S2.** Temporal similarity of comorbidity association patterns based on Jaccard coefficient across three admission periods.

| Comparison | N rules per period | Overlap \|A∩B\| | Union \|A∪B\| | Jaccard  similarity |
| --- | --- | --- | --- | --- |
| 2019-2020 vs 2021-2022 | 20 | 14 | 26 | 0.538 |
| 2019-2020 vs 2023-2024 | 20 | 6 | 34 | 0.176 |
| 2021-2022 vs 2023-2024 | 20 | 6 | 34 | 0.176 |

Jaccard index measures similarity between two sets, with the range 0-1 (0-no similarity, 1-same datasets).
