# Supplementary material for: Association rule mining and network analysis of the evolving comorbidity patterns in HIV inpatients in Baise, China
Source: Front Public Health. 2026 Mar 6;14:1717479. doi: 10.3389/fpubh.2026.1717479 (PMC13002846; doi:10.3389/fpubh.2026.1717479)
Supplement: Supplementary file 4 [file Table_4.docx]

**Table S4.** Basic network metrics of comorbidity networks across different admission periods.

| Admission Periods | Nodes | Edges | Density | Average degree |
| --- | --- | --- | --- | --- |
| 2019-2020 | 32 | 149 | 0.300 | 9.31 |
| 2021-2022 | 32 | 149 | 0.300 | 9.31 |
| 2023-2024 | 24 | 66 | 0.239 | 5.50 |
| 2019-2024 | 34 | 146 | 0.260 | 8.59 |
