# Supplementary material for: Association rule mining and network analysis of the evolving comorbidity patterns in HIV inpatients in Baise, China
Source: Front Public Health. 2026 Mar 6;14:1717479. doi: 10.3389/fpubh.2026.1717479 (PMC13002846; doi:10.3389/fpubh.2026.1717479)
Supplement: Supplementary file 6 [file Table_6.docx]

**Table S6.** Network-level characteristics based on the top 20 weighted edges across three admission periods.

| Admission periods | Nodes | Edges | Density | Average weighted degree | | Clustering coefficient |
| --- | --- | --- | --- | --- | --- | --- |
| 2019-2020 | 8 | 20 | 0.7143 | 1877.00 | 0.597 | |
| 2021-2022 | 7 | 20 | 0.9524 | 2643.43 | 0.613 | |
| 2023-2024 | 8 | 20 | 0.7143 | 848.50 | 0.409 | |
